# Evaluation of the Relationship Between Smoking and Insulin Resistance: A Case-Control Study

**DOI:** 10.7759/cureus.36684

**Published:** 2023-03-26

**Authors:** Aşkın K Kaplan, Yılmaz Sezgin

**Affiliations:** 1 Family Medicine, Maltepe University Faculty of Medicine, Istanbul, TUR; 2 Family Medicine, Istanbul Training and Research Hospital, Istanbul, TUR

**Keywords:** diabetes, cigarette smoking, serum lipid profile, vitamin b12, healthy subjects, insulin resistance, smoking tobacco

## Abstract

Introduction: In recent years, there has been a surge in research focusing on the link between smoking and insulin resistance in the context of obesity and diabetes. In this study, our objective was to investigate the relationship between smoking and insulin resistance.

Materials and Methods: This is a case-control study. The case and control groups were formed using the hospital patient information database and clinically randomized using data obtained, including age, gender, height, and weight. The case group for this study consisted of smokers, whereas the control group consisted of non-smokers. Chi-square tests were used to compare numbers and rates, and independent sample t-tests were used for the averages. Binary logistic regression analysis was performed between the case and control groups.

Results: According to logistic regression analysis, the odds ratio for non-smokers was 0.59 (0.31-1.14). The risk of insulin resistance is decreased by 41% non-significantly in non-smokers. The odds ratio for age was 1.03 (1.01-1.05). When the age variable increases by one unit, the risk of insulin resistance increase by 1.03 times.

Conclusion: Our study found no significant relationship between smoking and insulin resistance in healthy individuals. The relationship between smoking and insulin resistance, as reported in the scientific literature, may be suggestive of an association in which smoking exacerbates insulin resistance as a result of other contributing factors rather than serving as a direct causal factor. Further studies are warranted to elucidate the potential mechanisms underlying this association fully.

## Introduction

Tobacco smoke includes many chemical compounds [1]. Among these compounds, nicotine is the most prominent and well-established substance, which is responsible for the addictive effect of smoking [2]. Recently, there has been an increase in the number of studies investigating the association between smoking, obesity, and diabetes [3,4].

It is stated that nicotine is associated with type-2 diabetes by causing an increase in the glycemic index through autonomous nicotinic receptors [5]. In addition, although it has been suggested that nicotine plays a role in obesity and insulin resistance via leptin and adiponectin, the evidence has not been fully clarified [6]. Therefore, we aim to investigate the effects of smoking on insulin resistance.

## Materials and methods

Study design

This was designed to be a case-control study. The sample size was determined for logistic regression analysis, considering the number of five independent variables, such as age, sex, body mass index (BMI), smoking, and alcohol consumption. For each group, using the power analysis method, the minimum sample size of at least 42 was needed to detect a significant difference when taken into account at 0.05 type-I error (Alpha), 0.35 effect size, 0.80 power (1-beta).

Population selection

The case and control groups for this study were selected from the patient information database of Maltepe University Hospital, Istanbul, Türkiye, which included patients admitted between March 2020 and April 2021. The groups were matched based on age, gender, height, and weight criteria obtained from the database. The case group included 66 individuals with a smoking habit. The control group consisted of 150 individuals with non-smokers, matching the case group's gender, age, and BMI. The laboratory data of patients who were admitted to the family medicine outpatient during the last six months that met the inclusion criteria were analyzed. The study did not include patients with known diseases or those using medications.

Laboratory testing

At admission, blood samples were obtained from the study participants. Nine parameters, including glucose, insulin, thyroid stimulating hormone (TSH), cholesterol, triglyceride, high-density lipoprotein cholesterol (HDL-C), low-density lipoprotein cholesterol (LDL-C), vitamin D, and vitamin B12 were measured simultaneously for the analysis. Insulin resistance was defined using the homeostatic model assessment of insulin resistance (HOMA-IR) method. This involved calculating the product of fasting glucose (measured in mg/dL) and fasting insulin (measured in microU/mL), and dividing this value by a constant of 405. Higher HOMA-IR values indicate greater insulin resistance, with values above 2.7 considered indicative of insulin resistance.

Statistical analysis

All statistical analyzes were performed by using IBM SPSS Statistics for Windows, Version 25.0 (Released 2017; IBM Corp., Armonk, New York, United States). A value of P<0.05 was considered statistically significant. In the data expression, categorical data, numbers and percentages, and numerical data were expressed with averages and standard deviation. The distribution of demographic data was analyzed by frequency tests, categorical data comparison by chi-square test, and comparison of numerical data by independent sample t-test. The stepwise enter model was used in the binary logistic regression test to evaluate the effect of smoking on insulin resistance. Skewness and kurtosis analyses were used to conform the data to the normal distribution.

Ethical statement

The study did not require ethics committee approval because of retrospectively. The study was conducted retrospectively by collecting data using hospital information systems and patient records. All patients who applied to the hospital had signed an informed consent form that included information about the process and biochemical analyses to be conducted during their application and using their data. To ensure confidentiality, the data of each patient was transferred to the SPSS environment with a code, and all identification data were removed. The researchers followed all international conventions related to patient confidentiality and research ethics. All procedures performed in this study were by the ethical standards of the institutional and national research committee and with the 1964 Helsinki declaration and its later amendments or comparable ethical standards.

## Results

There was no difference between the case and control groups regarding alcohol consumption and BMI. However, the rate of women was higher in the control group than in the case group. In addition, the mean age was higher in the control group compared to the case group (Table 1).

**Table 1:** Comparison of the age, BMI, sex, and alcohol consumption between case and control groups *Statistical significance accepted as p <0.05 (2-tailed) SD: Standard Deviation, BMI: Body Mass Index

Characteristics of participants.		Case (n=66)	Control (n=150)	p-value
Age: Mean (SD)*		33.83 (12.57)	38.11 (15.16)	0.032
BMI: Mean (SD)		24.76 (4.32)	25.66 (4.77)	0.193
Gender: n (%)*	Female	34 (51.5)	106 (70.7)	0.006
	Male	32 (48.5)	44 (29.3)	
Alcohol consumption: n (%)	Yes	10 (15.2)	15.0 (10.0)	0.355
	No	56(84.8)	135 (90.0)	

Based on the independent sample t-test analysis, there was no difference in HOMA-IR values between smokers and non-smokers; additionally, significant differences were observed in terms of serum vitamin B12 levels, with the control group demonstrating higher vitamin B12 levels (Table 2). 

**Table 2:** Comparison of the serum biochemical parameters between case and control groups *İndependent simple t-test is significant at the P < 0.05 level (2-tailed). SD: Standard Deviation; HOMA-IR: Homeostatic Model Assessment for Insulin Resistance; HDL-C: High-Density Lipoprotein Cholesterol; LDL-C: Low-Density Lipoprotein Cholesterol; TSH: Thyroid-Stimulating Hormone

	Case (n=66)	Control (n=150)	t	p-value
	Mean ± SD			
Cholesterol (mg/dL)	188.85 ± 49.82	198.03 ± 44.97	-1.33	0.183
Triglyceride (mg/dL)	113.61 ± 54.75	112.28 ± 70.08	0.13	0.892
HDL-C (mg/dL)	52.39 ± 11.56	54.63 ± 11.32	-1.33	0.185
LDL-C (mg/dL)	116.03 ± 40.95	120.01 ± 39.20	-0.67	0.499
Glucose (mg/dL)	92.61 ± 12.76	94.75 ± 13.24	-1.10	0.270
Insulin (µU/mL)	8.30 ± 5.11	7.55 ± 5.22	0.96	0.333
TSH (mIU/L)	1.64 ± 1.15	1.89 ± 1.12	-1.51	0.132
Vitamin D (ng/mL)	17.48 ± 6.88	18.85 ± 10.23	-1.15	0.322
Vitamin B12 (ng/L)	160.35 ± 94.62	218.48 ± 117.35	-3.54	< 0.001*
HOMA-IR	1.91 ± 1.19	1.81 ± 1.43	0.45	0.647

Binary logistic regression analysis was performed considering the parameters that differ between smokers and non-smokers. The effects of smoking, age, sex, and vitamin B12 parameters on insulin resistance were analyzed. When solely evaluating smoking as an independent variable, the Nagelkerke R2 values were less than 0.2, indicating non-significant findings within the model. In addition, when other independent variables such as age, gender, and vitamin B12 are added, the model did not reach significance. As the Hosmer-Lemeshow test yielded a p-value greater than 0.05, the model's suitability of the model was considered satisfactory. However, changes in -2 Log Likelihood values in Step-1 and Step-2 (Chi-square, p > 0.05) did not reach significance. These results show that the logistic regression analysis is valid in general. As a result, our model predicts the outcome correctly with a probability of 69.9% (Table 3).

**Table 3:** Distribution of data showing the validity of the logistic regression analysis * Statistical significance accepted as p <0.05.

Method=Enter: Stepwise		-2 Log Likelihood	Omnibus Tests of Model Coefficients		Cox and Snell R Square	Nagelkerke R Square	Hosmer and Leweshow Test	Predicted percentage
			Chi-square	p				
Beginning		267.590						69.0
Step1	Smoking	264.620	3.049	0.081	0.014	0.020	0.207	69.0
Step2	+Age, Gender, Vitamin B12	256.478	1.525	0.217	0.050	0.070	0.256	69.9

According to logistic regression analysis, the odds ratio for non-smokers was 0.59 (0.31-1.14). The risk of insulin resistance is decreased by 41% in non-smokers. However, since p > 0.05, the difference did not reach statistical significance, and there was no difference between smokers and non-smokers. (Table 4). The odds ratio for age was 1.03 (1.01-1.05). When the age variable increase by one unit, the risk of insulin resistance increases by 1.03 times (Table 4).

**Table 4:** Logistic regression analysis showing the relationship between insulin resistance and variables of smoking, age, gender, and vitamin B12. ^a^Dependent Variable: Insulin resistance; *Logistic regression analysis significance  at p< 0.05 level (2-tailed) B: Estimated Coefficients; CI: Confidence Interval; SE: Standard Error

Insulin resistance ^a^		B	SE	Wald test	p	Risk (Odds) coefficient (Exp B)	95% CI for (Exp B)	
							Lower	Upper
Step1	Smoking (1)	-0.547	0.31	3.08	0.079	0.58	0.31	1.06
Step2	Smoking (1)	-0.527	0.35	2.48	0.115	0.59	0.31	1.14
	Age	0.027	0.01	6.21	0.013*	1.03	1.01	1.05
	Gender (1)	0.294	0.32	0.86	0.352	1.34	0.72	2.49
	Vitamin B12 (ng/L)	-0.002	0.01	1.41	0.998	0.99	0.99	1.01
	Constant	-1.32	0.47	7.93	0.005	0.27		

## Discussion

In the present study, we found no significant difference between the case and control groups regarding alcohol consumption status and BMI. However, we observed that the control group had a higher proportion of women and a higher mean age compared to the case group. These findings suggest that the randomization process may not have been sufficient to balance these factors between the groups. Nevertheless, we were able to overcome this limitation by performing regression analysis, which allowed us to adjust for potential confounding variables. Overall, our study highlights the importance of careful consideration and appropriate adjustments for confounding factors in order to obtain accurate and reliable results. As this is a retrospective study conducted in a single center, our findings may not be generalizable to other populations or healthcare settings, which can be considered a limitation of this study.

We found that serum vitamin B12 values ​​were lower in the case group compared to the control group. These findings support the existing knowledge in the literature. The observed lower serum vitamin B12 levels in the case group compared to the control group align with previous research. It has been well-established that smoking, a source of free radicals, is associated with decreased serum vitamin B12 levels [7,8]. The cyanide in tobacco smoke leads to an increase in serum cyanide levels, which in turn increases the excretion of thiocyanate from the kidneys and is linked to lower serum vitamin B12 levels [9]. The relationship between vitamin B12 levels and insulin resistance has been previously reported in non-diabetic obese individuals, with low levels of vitamin B12 being associated with insulin resistance [10]. Furthermore, a study has also demonstrated a relationship between vitamin B12 and insulin resistance in morbidly obese patients [11]. Although there are still some unclear findings regarding the relationship between vitamin B12 and type-2 diabetes [12], our study did not find any significant association between vitamin B12 and insulin resistance. This may suggest that the role of vitamin B12 in the development of insulin resistance and type-2 diabetes requires further investigation.

In our study, no significant difference was found between smokers and non-smokers in terms of HOMA-IR values, serum glycemic index, and lipid profile. According to regression analysis, the risk of insulin resistance is decreased by 41% in non-smokers, with smoking having a very low predictive power of only 2% on insulin resistance. However, since p > 0.05, this relationship is not statistically significant. Our findings are inconsistent with the literature. Only a few studies mention no relationship between smoking and insulin resistance in healthy people [13]. However, while smoking did not significantly affect insulin resistance in normal and overweight groups, it showed a significant increase in HOMA-IR in obese patients [13]. In a meta-analysis study, a significant association between passive smoking and type-2 diabetes was observed [14]. Additionally, a recent study found that smoking acts in synergy with genetic susceptibility to promote latent autoimmune diabetes in adults (LADA) [15].

All of this information suggests that the relationship between smoking and insulin resistance is not yet fully understood. It is known that nicotine stimulates catecholamine-mediated glucagon release from the adrenal medulla, which can increase gluconeogenesis and lead to hyperglycemia [4]. Therefore, the relationship between smoking and insulin resistance, frequently highlighted in the literature, may be a result rather than a cause. As a result, given the assumption that hyperinsulinemia-induced hypoglycemia episodes seen at the onset of type-2 diabetes can be prevented by increasing gluconeogenesis, nicotine may be preferred due to the avoidance behavior.

According to our study, insulin resistance increases with age. Age is associated with insulin resistance and mitochondrial muscle dysfunction, as also changes in body composition, which likely contribute to the development of age-related insulin resistance [16]. During aging, oxidative stress, intramyocellular lipid accumulation, the modified activity of insulin sensitivity regulatory enzymes, decreased autophagy, sarcopenia, mitochondrial dysfunction, and an over-activated renin-angiotensin system may occur. These modifications have the potential to negatively impact the insulin sensitivity of skeletal muscles and increase the risk of insulin resistance and type-2 diabetes during skeletal muscle aging [17].

## Conclusions

Our study provides evidence that there is no significant relationship between smoking and insulin resistance in healthy individuals. This may challenge the prevailing notion in the literature that smoking is a key risk factor for insulin resistance. Instead, it is possible that smoking and insulin resistance are both consequences of underlying pathophysiological mechanisms that have yet to be fully elucidated. Therefore, further studies involving detailed evaluations of healthy volunteers are needed to shed light on this complex relationship and inform clinical practice.
